# Clinical Sensitivity, Specificity and Epidemiology of SARS-CoV-2 Serological Testing Using the Biozek COVID-19 Test

**DOI:** 10.3390/diagnostics12010060

**Published:** 2021-12-28

**Authors:** Dennis Souverein, Theo G. Mank, Sjoerd M. Euser, Bjorn L. Herpers

**Affiliations:** Regional Public Health Laboratory Kennemerland, Department of Epidemiology, 2035 RC Haarlem, The Netherlands; t.mank@streeklabhaarlem.nl (T.G.M.); s.euser@streeklabhaarlem.nl (S.M.E.); b.herpers@streeklabhaarlem.nl (B.L.H.)

**Keywords:** SARS-CoV-2, COVID-19, epidemiology, validation, loss of smell

## Abstract

Background: Clinical validation using the Biozek COVID-19 test including sensitivity and specificity and associated patient-reported symptoms with SARS-CoV-2 seropositivity. Methods: 316 sera were analyzed including 47 hospitalized cases, 50 mild cases and 219 negative controls. Results were read visually by two technicians and in case of discrepancy by a third. Models were created between independent variables and IgG seropositivity using multivariable logistic regression analysis. Results: Sensitivity of both IgM and IgG together for hospitalized patients at all time periods was 68.1% (32/47) and 90.0% (27/30) after 10 days or more. From mild/asymptomatic cases the combined IgM and IgG sensitivity was 92.0% (46/50) and 91.8% (45/49) after 10 days or more. In the group of non-COVID-19 cases, the overall specificity was 99.1% (217/219). For IgG alone, the specificity was 99.5% (218/219). In the multivariable analysis loss of smell remained the strongest associated variable with an odds ratio (95%CI): 6.82 (5.61–8.31), *p*-value < 0.001. Our final prediction model yielded a ROC-AUC of 0.77 (0.74–0.81) showing acceptable discrimination. Conclusions: The Biozek COVID-19 test showed high specificity and good sensitivity 10 days after the first sickness day. Solely IgM positive tests must be interpreted with caution and preferably excluded. In order to capture most symptomatic COVID-19 cases, loss of smell should be included within symptomatic screening policies.

## 1. Introduction

The introduction of the SARS-CoV-2 virus and associated illness COVID-19 have resulted in a pandemic with disruption of traditional health systems and large social and economic impact [1,2]. Adequate surveillance and microbiological testing are important cornerstones within the bundle of interventions to monitor and control the spread of such outbreaks [3]. For SARS-CoV-2 there are broadly two options for microbiological testing, (1) detection of viral RNA within respirator samples and (2) detection of antibodies against SARS-CoV-2 [4]. Detection of SARS-CoV-2 RNA has a place within symptomatic patients to detect a causative agent, streamline medical management and justify isolation precautions [4]. Serological testing has two important places within microbiological testing, (1) patients who test PCR negative but are highly suspected for COVID-19 (progressive disease and/or typical CT-scan image) and (2) testing for antibodies within patients that had mild infection or were asymptomatic. Both are of importance as antibody presence may indicate that a person is not at risk for (re)infection, although this is not yet verified [5]. Therefore, serological testing may be of particular interest for healthcare workers (HCWs) and others who have an increased risk of infection due to their close contact with a COVID-19 patient [6]. On a population level, serological testing is important to follow the outbreak and estimate the population immunity level for SARS-CoV-2 which could provide support for the scaling down of nationwide control measures. Some studies analyzed associations between possible risk factors, symptoms and other patient-related factors and a SARS-CoV-2 immune response but were studied within the group of admitted (severe infected) patients excluding asymptomatic and/or mild cases [7,8,9]. To expand the knowledge on mild cases and associated pre-disposing risk factors and symptoms more studies are required.

In the present study, we are aiming to elaborate on multiple facets of SARS-CoV-2 serological testing using the Biozek COVID-19 test. In order to establish the clinical sensitivity and specificity of the Biozek COVID-19 test, serum samples from different patient groups such as hospitalized patients with severe clinical symptomatology due to COVID-19; patients with mild clinical symptomatology due to COVID-19, asymptomatic patients and patients with clinical symptomatology due to a non-COVID-19 etiology were tested. Secondly, in order to get more insight into the serodynamics, the Biozek COVID-19 test will be performed using sequential sera from proven SARS-CoV-2 infected patients and thirdly, the association between patient-reported symptoms, demographics and the serological status of (non-hospitalized) patients will be analyzed.

## 2. Materials and Methods

### 2.1. Test Characteristics and Procedures

The Biozek COVID-19 test (Biozek medical, Inzek B.V., Apeldoorn, The Netherlands) is a lateral flow immunochromatographic assay for the qualitative detection of SARS-CoV-2 IgG and IgM antibodies within serum, EDTA-blood or whole blood. All tests in this study were performed on serum. The testing of the Biozek COVID-19 test was conducted according to the manufacturer’s instructions. Test results were read visually by two technicians (observer-blind) and in case of discrepancy by a third, unaware of the initial findings.

### 2.2. Specimens for Calculating Clinical Sensitivity and Specificity of the Biozek COVID-19 Test

In total, 316 sera from unique patients were included in this validation. Of these, 47 sera were derived from hospitalized patients with a confirmed COVID-19 infection who tested PCR positive for SARS-CoV-2 from a respiratory sample. In addition, sera were derived from 50 non-admitted mild/asymptomatic cases that tested PCR positive. These 97 patients will be referred to as COVID-19 cases and their results will be considered the gold standard for the validation. For all COVID-19 cases, we also included the time between the first reported sickness day and the date of serum collection. In addition, 19 sera were derived from patients with a proven non-SARS-CoV-2 coronavirus (OC43, 229E, NL63, HKU1) infection (*n* = 12) and other respiratory infections (*n* = 7): Influenza A H1N1, Human metapneumovirus, Rhinovirus and Mycoplasma pneumoniae. Besides these sera from patients with acute respiratory infections, 10 random sera derived from patients and subsequently stored in 2019 were added. In 2019, no SARS-CoV-2 patients were reported in the Netherlands, so the samples of these patients can be designated as true negatives. Furthermore, 190 samples of PCR negative patients were included. In total, 219 patients will be referred to as non-COVID-19 cases, and their samples will be considered as the gold standard population for the specificity analysis.

### 2.3. Specimens for Analyzing Sequential Samples of PCR Positive Persons

From 25 PCR positive persons, sequential serum samples were collected in order to analyze their serodynamic using the Biozek COVID-19 test. All patients were hospitalized and classified as severe cases. For every patient, the date of onset of symptoms and test results were registered. For every patient, the difference between the test date and onset of symptoms was calculated. Through the dynamic design of this study, the number of days was different for every patient. Missing days between the minimum and maximum known results were filled by the previous known entry of each patient. Outside the minimum and maximum, no filling was performed.

### 2.4. Specimens for Analyzing the Association between Patient-Reported Symptoms between Negative and Seropositive (Non-Hospitalized) Patients

For this part of the study, an anonymized dataset of Labonovum, a Direct-to-consumer (DTC) laboratory was used. The Biozek COVID-19 test was used as described within this methods section. In total, from 3486 non-hospitalized persons, serological test results, as well as a short questionnaire, were obtained. This questionnaire included (1) person demographics, (2) symptoms and (3) possible risk factors. When patients had no symptoms but requested a test, the date of onset of symptoms was not registered. Sera were self-collected by finger prick as described earlier using Hem-Col which is a novel blood collection device that is designed to collect capillary blood drawn with a finger prick [10]. Hem-Col is a microtube containing an anticoagulant and a preservation buffer to enhance analyte stability in whole blood. Hem-Col samples were sent by post to Labonovum, and the questionnaire was taken at the time of online registration with the person’s consent. For the association analysis patients were classified in two categories: (1) IgM and IgG negative and (2) IgG positive (all patients with no first sickness day reported) or when reported only after 14 days between serum collection date and the onset of symptoms.

### 2.5. Statistical Analysis

For the comparison of sensitivity and specificity percentages, the McNemar’s test was used (*p*-value < 0.05 was considered statistically significant). The 95% confidence intervals were calculated using the Score confidence interval (when the proportion was in the range (5–95%), or with the exact confidence interval. The association and prediction model between patient-reported symptoms and SARS-CoV-2 seropositivity were analyzed using (multivariable) logistic regression analysis. All statistical analyses were performed using R version 3.6.1, Vienna, Austria.

## 3. Results

### 3.1. Validation Biozek COVID-19 Test

A total of 47 sera from severe, hospitalized COVID-19 cases were tested with the Biozek COVID-19 test (Table 1). The calculated sensitivity of both IgM and IgG together at all time periods was 68.1% (32/47) (Table 2). The mean [range] number of days between first sickness day and testing was 14 (2–90). At day 10 or higher 90.0% (27/30) tested IgG positive. From mild/asymptomatic cases 50 sera were tested. The combined IgM and IgG sensitivity was 92.0% (46/50). The mean [range] number of days between first sickness day and testing was 50 (0–117). On day 10 or higher 91.8% (45/49) tested IgG positive. Combining severe and mild/asymptomatic cases together we found a sensitivity for IgM and IgG together of 80.4% (78/97).

A total of 219 sera from non-COVID-19 cases were tested with the Biozek COVID-19 test. None of the 12 (non-SARS-CoV-2) coronavirus-positive patients and seven other respiratory infections tested positive for IgM or IgG. One out of all sera tested was IgM positive. In the group of non-COVID-19 cases, the overall specificity was 99.1% (217/219). For IgG alone, the specificity was 99.5% (218/219).

### 3.2. Serodynamics of PCR Positive Persons

**Figure 1:**
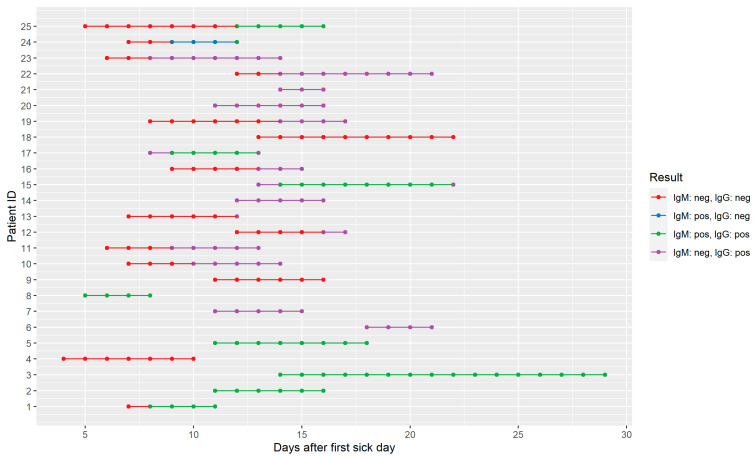
Individual sequential serological results of the Biozek COVID-19 test.

**Figure 2:**
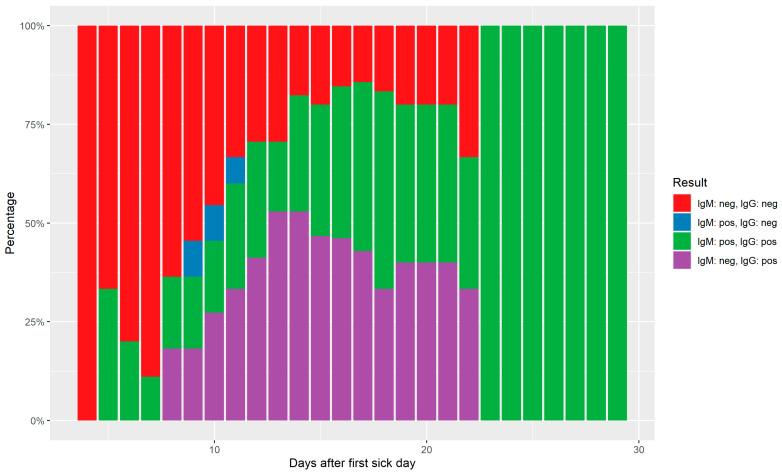
Relative aggregated sequential serological results over time.

From 25 PCR confirmed cases, consecutive serum samples were collected and tested with the Biozek COVID-19 test. All samples were collected randomly after the first sickness day. The first sample was collected on day four and this patient tested negative. On day 15, 80% (12/15) of the patients tested IgG positive. Figure 1 shows the individual patient patterns and Figure 2 the relative frequencies of all aggregated results over time.

### 3.3. Association Model between Patient Reported Symptoms and Seropositivity

Table 3 shows the association between IgG seropositivity, and all included independent variables. First, all independent variables were analyzed univariably. Variables that were significantly associated with seropositivity were fever, short breath, loss of smell, itchy skin, tiredness, contact with a person with influenza-like symptoms, being present on a location with many people and recent travel to a foreign country. The strongest associated variable was the loss of smell, odds ratio (95% CI): 6.20 (3.96–11.47), *p*-value < 0.001. Sneezing, sore throat and abdominal pain were negatively associated with seropositivity. Abdominal pain was the strongest negatively associated variable, odds ratio (95% CI): 0.63 (5.26–7.32), *p*-value < 0.001.

The univariable associations did not markedly change after adjustment for possible confounders using multivariable logistic regression analysis. The strongest association remained loss of smell, odds ratio (95% CI): 6.82 (5.61–8.31), *p*-value < 0.001. Short breath was univariable associated with seropositivity, odds ratio (95% CI): 1.29 (1.11–1.50), *p*-value = 0.001 and flipped around in the multivariable analysis, odds ratio (95% CI): 0.82 (0.67–0.99), *p*-value = 0.045. All other results are found in Table 3.

### 3.4. Prediction Model for SARS-CoV-2 Seropositivity

**Figure 3:**
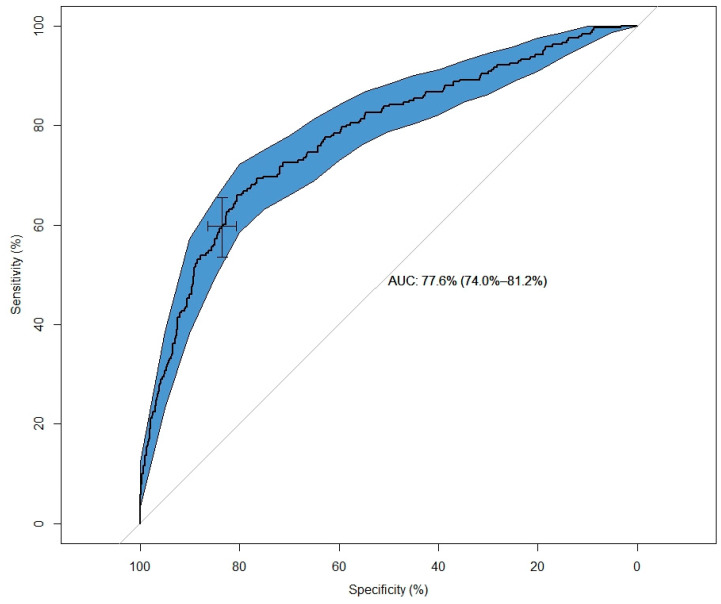
ROC for prediction of an IgM positive serological test.

All variables that were used in the association model were used to build a prediction model using a backward selection procedure with an inclusion criterium of *p* < 0.05 for all remaining variables. The included variables were age, sex, fever, sneezing, sore throat, loss of smell, abdominal pain, contact with someone with ILI symptoms and recent travel to a foreign country. After this reduction step we split the total dataset into a 75% training (*n* = 2615) and 25% validation (*n* = 871) set. After running the final model on the validation set, we found a ROC-AUC of 0.77 (0.74–0.81), a sensitivity of 0.60 (0.54–0.66), a specificity of 0.83 (0.80–0.86) for the highest Youden index at a threshold of 0.32 (Figure 3).

## 4. Discussion

In the present study, we showed that the Biozek COVID-19 test had a high specificity of 99.1% for the detection of antibodies against SARS-CoV-2. We found no false positives against other respiratory pathogens including other coronaviruses. As expected, the overall sensitivity was low as we also included sera that were obtained shorter than 10 days between the first reported sickness day and sera collection. After 10 days, the sensitivity was 90.0% for severe cases. For mild/asymptomatic cases, the sensitivity was 92.0% and 91.8% after 10 days. Consecutive serological test results of cases revealed that the first positive test results were found on day 5 and that solely IgM positive persons were not found after day 11.

From our validation data, it appears that solely low positive IgM is associated with false positives. We found no additional benefit of solely IgM combined with IgG when interpreting seropositivity. Other studies that validated lateral flow serological tests found overall sensitivities of around 70% increasing above 90% after the second week and specificities reaching 100% [11,12]. These results are in line with our validation. We showed in our study that IgM does not increase the sensitivity significantly. Generally, IgM is produced first followed by a switch towards IgG production, but some studies suggest that IgM and IgG develop around the same time [13,14]. This means that IgM is not very discriminative in addition to IgG. For the Biozek COVID-19 test, we advise interpreting solely low positive IgM as negative to avoid false positives and increase positive predicted values.

Serology tests can be performed on several samples like whole blood, serum and EDTA-blood. From the literature, it is known that the test characteristics are dependent on sample type. Serum samples are considered the best sample type for serological testing, but a study showed no difference in different types of blood samples [15]. In the present study, we only tested serum samples and do not recommend whole blood samples or testing outside the controlled setting of the laboratory as this can possibly produce unreliable test results.

PCR testing offers a rapid detection of SARS-CoV-2 but can only be detected during viral shedding. Several studies indicated that 21 days after symptom onset 50% of the cases achieved viral clearance. Serological testing is a valuable strategy in highly suspected or convalescent cases where no causative pathogen can be detected [4]. Outside the clinical test setting, serological testing has also a place on a population level in order to estimate the population seroprevalence and/or herd immunity [4]. Several studies reported these prevalences. Spain reported a mean seroprevalence of 5% with substantial regional differences with above 10% within Madrid and lower (<3%) in coastal areas [16]. In the Netherlands, similar results are reported with a mean nationwide prevalence of 5.4% and regional differences showing higher prevalences (>10%) in the south Netherlands compared to north Netherlands (<3%) [17]. Whether the detection of antibodies provides immunity differs from one pathogen to another [18]. For COVID-19, this is largely unknown. This complicates the application of widespread serological testing and assumptions about immunity, re-infection and transmission when working without PPE. Especially within healthcare settings, where PPE was scarce during the peak of the pandemic.

In the group of severe cases, we found that at day 10 after symptom onset, 90.0% (27/30) tested IgG positive. In comparison, for MERS, studies found that all surviving patients seroconverted within three weeks and 93% with a mean time of 20 days for SARS [19,20]. It looks like the antibody response for SARS-CoV-2 is similar and possibly earlier compared to these other coronaviruses.

The Biozek COVID-19 test was tested in a larger population including a short questionnaire before testing. Questions consisted of person demographics, symptoms and possible predisposing risk factors. We built a multivariable association model and found that loss of smell and fever were the strongest associated symptoms with an odds ratio (95%CI) of 6.82 (5.61–8.31) for loss of smell and 1.70 (1.40–2.07) for fever. Loss of smell seems to be an important discriminating factor. Menni et al. found in a similar study an odds ratio (95% CI) of 6.74 (6.31–7.21) and that including loss of smell within symptom-based identification algorithms added 16% above fever and cough alone [21]. Combining loss of smell, cough and fever enabled them to identify 87.5% of the symptomatic patients [22]. In order to capture most symptomatic COVID-19 cases, we encourage including the loss of smell within symptomatic screening policies. Our prediction model showed an AUC of 0.77, which could be interpreted as acceptable following the criteria of Hosmer and Lemeshow [23]. In line with our estimated odds ratios, the discrimination power of our prediction model was nearly identical in comparison with Menni et al. reporting an AUC of 0.76.

The present study has some important strengths and limitations. Our study alone is not sufficient to validate the Biozek COVID-19 test. More studies with the inclusion of several populations and larger sample sizes are needed. Especially for the validation of specificity samples with all kinds of other (respiratory) infections and possible disruptive factors, such as rheumatic patients need to be included. The strong part of the validation is the inclusion of samples from asymptomatic/mild cases. Another strong point of this study is the inclusion of serological testing in a large population of persons who are asymptomatic or had mild symptoms including questions regarding symptoms. This enabled us to estimate associations between symptoms and detection of antibodies. Although we did not have the PCR status of these patients, we have confidence in the results of these tests. In order to exclude possible false-positive results, we have excluded solely IgM positive persons within this part of the study.

In conclusion, the Biozek COVID-19 test showed high specificity and good sensitivity 10 days after the first sickness day. Solely IgM positive tests must be interpreted with caution and preferably excluded. In order to capture most symptomatic COVID-19 cases loss of smell should be included within symptomatic screening policies.

## Figures and Tables

**Table 1:** Results of the Biozek COVID-19 test.

	IgM		IgG		Total
	Pos	Neg	Pos	Neg	
Severe cases	12	35	31	16	47
Mild/asymptomatic cases	5	45	46	4	50
**Total cases**	17	80	77	20	97
Random negative controls	1	9	0	10	10
Other acute respiratory infections	0	7	0	7	7
Non-SARS-CoV-2 corona infections	0	12	0	12	12
Negative SARS-CoV-2 PCR patients	0	190	1	189	190
**Total controls**	1	218	1	218	219

**Table 2:** Sensitivity and specificity of the Biozek COVID-19 test.

	Mean Number of Days between First Sick Day and Testing (Range)	Sensitivity	Sensitivity (≥10 Days)	Specificity
Severe cases	14 (2–90)			
IgM		25.5%	30.0%	99.5%
IgG		66.0%	90.0%	99.5%
IgM and IgG		68.1%	90.0%	99.1%
Mild/asymptomatic cases	50 (0–117)			
IgM		10.0%	10.2%	99.5%
IgG		92.0%	91.8%	99.5%
IgM and IgG		92.0%	91.8%	99.1%
Combined severe and asymptomatic cases	32 (0–117)			
IgM		17.5%	17.7%	99.5%
IgG		79.4%	91.1%	99.5%
IgM and IgG		80.4%	91.1%	99.1%

**Table 3:** Comparison between IgM, IgG negative persons (first day of symptoms longer than 14 days) compared to IgG positive persons.

Variable		IgM: Neg & IgG: Neg	IgG: Pos	Total	OR (Univariable)	OR (Multivariable)
Age	Mean (SD)	45.6 (15.1)	48.3 (13.7)	46.3 (14.8)	1.01 (1.01–1.02, *p* < 0.001)	1.02 (1.01–1.03, *p* < 0.001)
Time between onset of symptoms and testing	Mean (SD)	48.4 (22.8)	39.2 (15.0)	46.0 (21.4)	0.97 (0.97–0.98, *p* < 0.001)	0.97 (0.96–0.97, *p* < 0.001)
Sex	Female	1365 (52.4)	428 (48.7)	1793 (51.4)		
	Male	1242 (47.6)	451 (51.3)	1693 (48.6)	1.16 (0.99–1.35, *p* = 0.060)	1.19 (0.99–1.44, *p* = 0.068)
Fever	No	1513 (58.0)	345 (39.2)	1858 (53.3)		
	Yes	1094 (42.0)	534 (60.8)	1628 (46.7)	2.14 (1.83–2.50, *p* < 0.001)	1.70 (1.40–2.07, *p* < 0.001)
Sneezing	No	1510 (57.9)	559 (63.6)	2069 (59.4)		
	Yes	1097 (42.1)	320 (36.4)	1417 (40.6)	0.79 (0.67–0.92, *p* = 0.003)	0.70 (0.57–0.85, *p* < 0.001)
Runny nose	No	932 (35.7)	324 (36.9)	1256 (36.0)		
	Yes	1675 (64.3)	555 (63.1)	2230 (64.0)	0.95 (0.81–1.12, *p* = 0.553)	0.87 (0.71–1.07, *p* = 0.197)
Sore throat	No	1038 (39.8)	442 (50.3)	1480 (42.5)		
	Yes	1569 (60.2)	437 (49.7)	2006 (57.5)	0.65 (0.56–0.76, *p* < 0.001)	0.48 (0.39–0.59, *p* < 0.001)
Short breath	No	1429 (54.8)	426 (48.5)	1855 (53.2)		
	Yes	1178 (45.2)	453 (51.5)	1631 (46.8)	1.29 (1.11–1.50, *p* = 0.001)	0.82 (0.67–0.99, *p* = 0.045)
Loss of smell	No	2016 (77.3)	312 (35.5)	2328 (66.8)		
	Yes	591 (22.7)	567 (64.5)	1158 (33.2)	6.20 (5.26–7.32, *p* < 0.001)	6.82 (5.61–8.31, *p* < 0.001)
Itchy skin	No	2308 (88.5)	769 (87.5)	3077 (88.3)		
	Yes	299 (11.5)	110 (12.5)	409 (11.7)	1.10 (0.87–1.39, *p* = 0.405)	1.07 (0.81–1.42, *p* = 0.617)
Diarrhea	No	1692 (64.9)	539 (61.3)	2231 (64.0)		
	Yes	915 (35.1)	340 (38.7)	1255 (36.0)	1.17 (1.00–1.37, *p* = 0.056)	1.14 (0.93–1.40, *p* = 0.209)
Abdominal pain	No	1820 (69.8)	690 (78.5)	2510 (72.0)		
	Yes	787 (30.2)	189 (21.5)	976 (28.0)	0.63 (0.53–0.76, *p* < 0.001)	0.58 (0.46–0.73, *p* < 0.001)
Headache	No	867 (33.3)	246 (28.0)	1113 (31.9)		
	Yes	1740 (66.7)	633 (72.0)	2373 (68.1)	1.28 (1.08–1.52, *p* = 0.004)	1.12 (0.90–1.40, *p* = 0.323)
Tired	No	548 (21.0)	110 (12.5)	658 (18.9)		
	Yes	2059 (79.0)	769 (87.5)	2828 (81.1)	1.86 (1.50–2.33, *p* < 0.001)	1.04 (0.78–1.40, *p* = 0.789)
Contact with ILI	No	1275 (48.9)	334 (38.0)	1609 (46.2)		
	Yes	1332 (51.1)	545 (62.0)	1877 (53.8)	1.56 (1.34–1.83, *p* < 0.001)	1.46 (1.21–1.76, *p* < 0.001)
Location with many people	No	774 (29.7)	220 (25.0)	994 (28.5)		
	Yes	1833 (70.3)	659 (75.0)	2492 (71.5)	1.26 (1.06–1.51, *p* = 0.008)	1.16 (0.93–1.45, *p* = 0.182)
Foreign country	No	1700 (65.2)	478 (54.4)	2178 (62.5)		
	Yes	907 (34.8)	401 (45.6)	1308 (37.5)	1.57 (1.35–1.84, *p* < 0.001)	1.67 (1.38–2.04, *p* < 0.001)
Which foreign country	No	1700 (65.2)	478 (54.4)	2178 (62.5)		
	Belgium	16 (0.6)	7 (0.8)	23 (0.7)	1.56 (0.60–3.67, *p* = 0.332)	
	Germany	48 (1.8)	7 (0.8)	55 (1.6)	0.52 (0.21–1.08, *p* = 0.107)	
	France	55 (2.1)	25 (2.8)	80 (2.3)	1.62 (0.98–2.59, *p* = 0.052)	
	Italy	109 (4.2)	29 (3.3)	138 (4.0)	0.95 (0.61–1.42, *p* = 0.797)	
	Austria	328 (12.6)	247 (28.1)	575 (16.5)	2.68 (2.21–3.25, *p* < 0.001)	
	Spain	78 (3.0)	19 (2.2)	97 (2.8)	0.87 (0.51–1.41, *p* = 0.582)	
	Other European countries	103 (4.0)	29 (3.3)	132 (3.8)	1.00 (0.64–1.51, *p* = 0.995)	
	Africa	40 (1.5)	12 (1.4)	52 (1.5)	1.07 (0.53–1.99, *p* = 0.846)	
	China	20 (0.8)		20 (0.6)		
	Middle East	24 (0.9)	7 (0.8)	31 (0.9)	1.04 (0.41–2.30, *p* = 0.933)	
	North America	49 (1.9)	13 (1.5)	62 (1.8)	0.94 (0.49–1.70, *p* = 0.854)	
	South America	27 (1.0)	5 (0.6)	32 (0.9)	0.66 (0.22–1.58, *p* = 0.394)	
	Australia	10 (0.4)	1 (0.1)	11 (0.3)	0.36 (0.02–1.86, *p* = 0.325)	

## Data Availability

The data underlying this article will be shared on reasonable request to the corresponding author.
